# Etiology and Outcomes in Patients With Chronic Kidney Disease and Ascites

**DOI:** 10.7759/cureus.64113

**Published:** 2024-07-08

**Authors:** Gollapudi Sai Spandana, Stalin Viswanathan, Deepak Barathi S, Jayachandran Selvaraj

**Affiliations:** 1 Nephrology, Jawaharlal Institute of Postgraduate Medical Education and Research, Pondicherry, IND; 2 General Medicine, Jawaharlal Institute of Postgraduate Medical Education and Research, Pondicherry, IND; 3 Radiology, Jawaharlal Institute of Postgraduate Medical Education and Research, Pondicherry, IND

**Keywords:** outcomes, etiology, nephrogenic ascites, ascites, chronic kidney disease

## Abstract

Introduction

Nephrogenic ascites is an uncommon disorder associated with grave prognosis. Studies on etiopathogenesis and outcomes are scarce. This study aimed to identify the etiologies of ascites in patients with chronic kidney disease (CKD) and estimate the proportion of nephrogenic ascites and the 90-day mortality.

Methods

This was a prospective, observational, and hospital-based study. Consecutive patients with CKD admitted to a tertiary care government teaching hospital were recruited. History, examination, investigations, and evaluation of the etiology of ascites were performed. Ascites was classified into high and low serum albumin-ascites gradient types. Patients with ascites were also followed up for three months to monitor for worsening symptoms, further workup (if necessary), and mortality.

Results

A total of 355 patients were recruited, with 72.5% being males. Of these, 76 were newly diagnosed with CKD. The most common comorbidities were diabetes mellitus and hypertension. Forty patients had ascites with a mean duration of CKD and hemodialysis of 20.9±23.1 months and 9.3±15.5 months, respectively. Thirteen of the 40 patients with ascites were lost to follow-up. Among the remaining 27, 13 died during follow-up. A known etiology was seen in 29 of the 40 (72.5%) patients. The multiple etiologies group (any combination of cardiac or liver disease, malignancy, and hypothyroidism) constituted 21 patients. Overall, among the 40 patients with ascites, 11 (27.5%) had nephrogenic ascites of whom, four died during follow-up.

Conclusions

Nephrogenic ascites was observed in 11 patients. Most patients with ascites in CKD have an identifiable etiology. The prognosis of ascites in patients with CKD in our study was dismal.

## Introduction

Chronic kidney disease (CKD) is a leading cause of death and disability worldwide. In 2016, CKD was listed as the 13th leading cause of death worldwide, and by 2040, it is expected to be the fifth leading cause of years of life lost [1]. In 2017, the global prevalence of CKD was 11.1% (10.4% among men and 11.8% among women), amounting to an absolute global number of 843.6 million [1]. The prevalence of CKD among Indian adults in 2013 was 17.2%, according to the Screening and Early Evaluation of Kidney diseases (SEEK) study [2]. A systematic review of the prevalence of CKD in South Asia reported a pooled prevalence of 10.2% among Indian adults [3].

The association between ascites and CKD was first reported in 1970 in two separate studies [4,5]. Since then, a few case reports and case series have tried to explore this entity further [6-16]. The term ‘nephrogenic ascites’ is defined as clinically evident refractory ascites of unknown etiology that occurs in patients with end-stage renal disease (ESRD), many of whom may be undergoing hemodialysis [6-9]. Other synonyms include nephrogenous ascites, hemodialysis-associated ascites, dialysis ascites, ascites associated with ESRD, and idiopathic ascites [6]. However, the term ‘nephrogenic ascites’ is preferred because the onset of ascites may sometimes precede the initiation of dialysis. However, its pathogenesis has not been well established. A multitude of factors have been implicated in its formation [6-9]. Nephrogenic ascites is usually associated with grave prognosis, with an average survival of 7-10.7 months [6,7]. 

Ascites in patients with CKD present a complex diagnostic and therapeutic clinical challenge. However, the literature pertaining to its etiology, pathophysiology, treatment, and prognosis is scarce. There is limited prospective data on this entity from Asia and the Indian subcontinent. In this study, the authors attempted to identify the different etiologies contributing to ascites in patients with advanced-stage CKD and to estimate the magnitude of patients with nephrogenic ascites. 

This article was previously posted to the Research Square preprint server on May 19, 2023.

## Materials and methods

This was a prospective, hospital-based observational study conducted at Jawaharlal Institute of Postgraduate Medical Education and Research, Pondicherry, India, between March 1, 2021, and December 31, 2022. Consecutive patients aged ≥18 with CKD stage ≥ 3 were recruited for this study. Patients treated for malignancy and tuberculosis were excluded, as were patients on continuous ambulatory peritoneal dialysis (CAPD) and those who had previously worked up for ascites. CKD was diagnosed according to the 2012 KDIGO guidelines [17]. This study was approved by the Institutional Ethics Committee for Observational Studies, Jawaharlal Institute of Postgraduate Medical Education and Research (approval number: JIP/IEC/2020/319). All methods were performed following relevant guidelines and regulations. Written informed consent was obtained from all the patients.

Data were collected using a mobile application (Epicollect5; The Centre for Genomic Pathogen Surveillance, University of Oxford, Oxford, United Kingdom). Demographic variables, clinical history, comorbidities, CKD, dialysis-related factors, and treatment histories were recorded. Vital signs, abdominal examination for shifting dullness or fluid thrill, and other systemic examinations were recorded. Biochemistry, hematology, urinalysis (except in anuric patients), electrocardiography, ECG, chest radiography, and ultrasonography were also performed. The sample size was based on an assumed prevalence of nephrogenic ascites of 30%, yielding a sample size of 355, since studies have shown prevalence rates ranging from 15% to 77% [16,18].

Patients with moderate-to-severe ascites underwent paracentesis, and the fluid was analyzed for biochemistry, cytology, and microbiology (Gram stain, culture, and GenExpert (Cepheid India Pvt. Ltd, Bangalore, India)). Serum albumin ascitic fluid albumin (SAAG), was calculated, and patients with ascites were classified into low or high SAAG groups using an SAAG cut-off of ≥1.1 [19]. Patients in each group were further sub-classified as having low or high ascitic fluid protein (AFP) using an ascitic fluid total protein cutoff ≥ 2.5 g/dL [19,20]. Mixed ascites occurs when portal hypertension is additionally associated with non-portal-hypertension-related (low-SAAG) causes [20]. Additional testing in patients with ascites included thyroid function tests, serum amylase, and lipase levels, serology for hepatitis B and C, ultrasonography of the abdomen for liver echotexture and features of portal hypertension, two-dimensional (2D) echocardiography for evidence of right and left heart failure, and abdominal CT (in some cases). Patients whose etiology of ascites remained unclear despite initial workup were maintained on follow-up for three months to monitor for the persistence of symptoms, recurrence of new symptoms, treatment initiation and compliance, increased frequency of dialysis sessions, or any increase in diuretic therapy dosage.

Statistical analysis

The data were analyzed using IBM SPSS Statistics for Windows, Version 22.0 (Released2013; IBM Corp., Armonk, New York, United States). Descriptive and inferential statistics were used to analyze the data. Categorical variables were reported as frequencies with percentages. Continuous data were first tested for normality of distribution using the Kolmogorov-Smirnov test and then summarized as mean±SD) or median (IQR) based on their distribution. Categorical variables were compared using the chi-square test. The strength of the association was measured using the odds ratio. The two-tailed unpaired t-test was used to compare normally distributed continuous data, whereas the Mann-Whitney U test was used for non-normally distributed continuous data. For all inferential statistical tests, a p-value of ≤0.05 was considered statistically significant.

## Results

**Figure 1:**
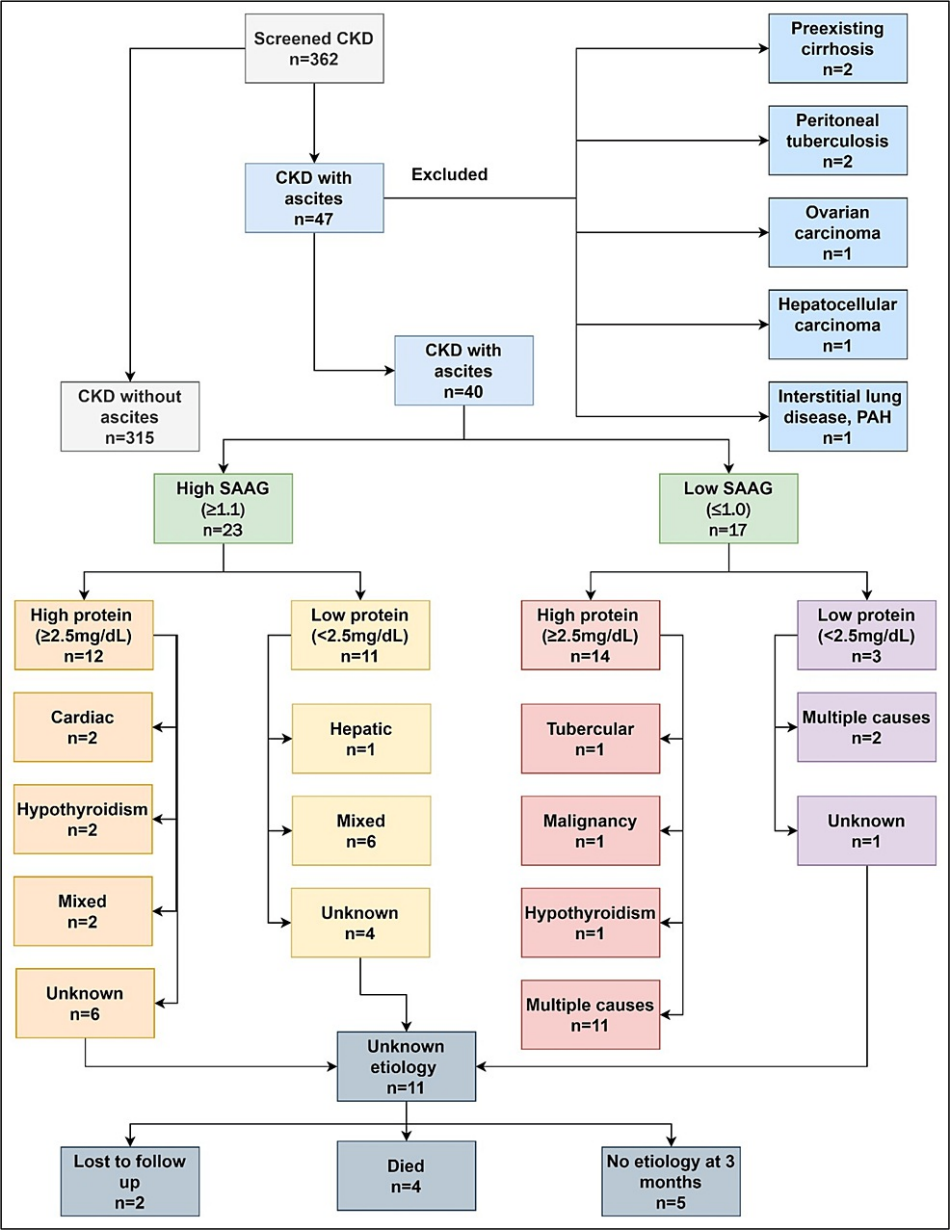
Flow chart depicting the study population, classification and etiology of ascites. CKD: chronic kidney disease; SAAG: serum ascites-albumin gradient; PAH: pulmonary artery hypertension

A total of 355 (among 362 screened) patients with CKD were recruited with a mean age of 46.9 ± 13.3 years. Seven patients were excluded because they already had a known etiology of ascites (Figure 1).

The mean duration of CKD was 24.3±35 months. A functional arteriovenous fistula (AVF) was observed in 69 (50%) patients on MHD. Hypertension and diabetes mellitus were the most common comorbidities (Table 1).

**Table 1:** Baseline characteristics of the entire study population (N=355) CKD: chronic kidney disease; JVP: jugular venous pulse

Demography and comorbidities		Values
Age (years), mean±SD		46.9 ± 13.3
Males, n (%)		260 (72.5)
Newly diagnosed CKD, n (%)		76 (21.4)
CKD on dialysis, n (%)		138 (38.8)
Diabetes mellitus, n (%)		115 (32.4)
Hypertension, n (%)		251 (70.7)
Coronary artery disease, n (%)		24 (6.8)
Heart failure, n (%)		12 (3.4)
Chronic liver disease, n (%)		5 (1.4)
Past tuberculosis (n)		17 (4.8)
Renal transplant recipients (n)		4 (1.1)
Smoking history, n (%)		121 (34.1)
Alcohol consumption, n (%)		152 (42.8)
Symptoms, n (%)	Abdominal distension	68 (19.2)
	Fever	107 (30.1)
Signs, n (%)	Hypertensive crisis (≥180/≥120mmHg)	75 (21.1)
	Elevated JVP	146 (41.1)
	Anasarca	16 (4.5)
	Pericardial rub	9 (2.5)
	Flapping tremors	66 (18.6)
Chest radiography, n (%)	Cardiomegaly	45 (12.7)
	Pleural effusion	72 (20.3)
	Pulmonary edema	105 (29.6)
Electrocardiography, n (%)	Left ventricular hypertrophy	131 (36.9)
Echocardiography, n (%)	Left ventricular hypertrophy	135 (45)
	Pericardial effusion	30 (10)
Ultrasonography, n (%)	Significant ascites	40 (11.3)

**Figure 2:**
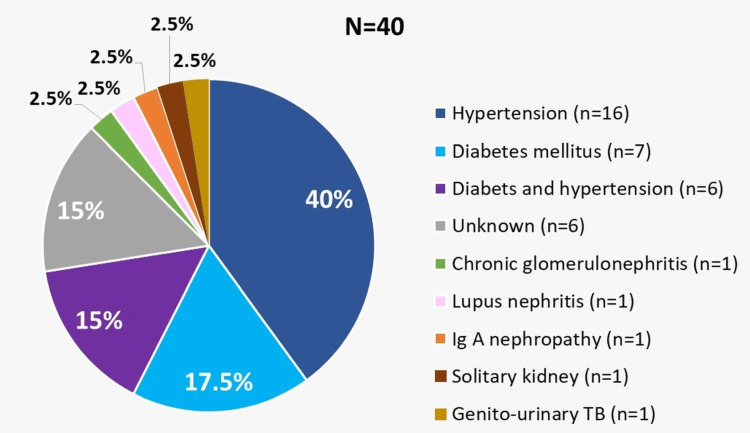
The etiology of chronic kidney disease in patients with ascites TB: tuberculosis

The etiology of CKD in patients with ascites is given in Figure 2. Forty patients (11.3%) had significant ascites of which six (15.0%) were newly diagnosed with CKD. Of the remaining 34 patients, 23 (57.5%) received MHD. Eight (20%) had features of liver cirrhosis on ultrasound, 19 (47.5%) patients had heart failure with reduced ejection fraction, eight (20%) had elevated right ventricular systolic pressures, and five had pericardial effusion on echocardiography.

Chest radiography revealed cardiomegaly in 15 patients (37.5%) and pleural effusion in 18 (45%). Electrocardiography showed left ventricular hypertrophy (LVH) in 12 (30%) and low-voltage complexes in four (10%). A comparison of the patients with and without ascites is presented in Table 2.

**Table 2:** Comparison of CKD patients with and without ascites. CKD: chronic kidney disease; MHD: maintenance hemodialysis; TSH: thyroid stimulating hormone

Demography and Co-morbidities	Ascites group (N=40)	Non- ascites group (N=315)	OR (95% CI)	P Value
Age (years), mean±SD	46.5 ± 11.0	47.0 ± 13.5	-0.48 (-4.86 to 3.90)	0.82
Duration of CKD (months), mean±SD	20.9 ± 23.1	24.8 ± 36.2	-3.90 (-15.47 to 7.66)	0.50
Duration on MHD (months), mean±SD	9.3 ± 15.5	5.1 ± 12.3	4.23 (0.35 to 8.44)	0.04
Diabetes mellitus, n (%)	13 (32.5)	102 (32.4)	1.00 (0.49 to 2.0)	0.98
Hypertension, n (%)	27 (67.5)	224 (71.1)	0.84 (0.41 to 1.70)	0.63
Coronary artery disease, n (%)	3 (7.5)	21 (6.7)	1.13 (0.32 to 3.99)	0.84
Past tuberculosis, n (%)	1 (2.5)	39 (97.5)	0.47 (0.06 to 3.71)	0.47
Renal transplant recipient, n (%)	1 (2.5)	3 (1.0)	2.77 (0.27 to 26.26)	0.38
Past blood transfusions, n (%)	8 (20.0)	18 (5.7)	4.12 (1.66 to 10.23)	0.001
Significant weight loss, n (%)	5 (12.5)	9 (2.9)	4.85 (1.54 to 15.3)	0.003
Arteriovenous fistula, n (%)	11 (27.5)	58 (18.4)	1.68 (0.79 to 3.55)	0.17
Serum albumin (g/dL), mean±SD	2.6 ± 0.7	3.3 ± 1.7 (n=297)	-0.66 (-1.22 to -0.11)	0.001
Serum TSH (mIU/ml), mean±SD	6.2 ± 5.7 (n=29)	4.7 ± 11.5 (n=87)	1.5 (-2.92 to 5.93)	0.81

Of the 40 patients with significant ascites, a known contributing factor(s) for ascites was identified in 29 (72.5%) patients. Based on history, clinical examination, baseline investigations, and imaging, it was found that a significant proportion of patients had cardiac or multiple causes (≥1 etiology) of ascites; 23% had high-SAAG ascites (Figure 1).

The remaining 11 patients with an unknown etiology could be ascribed to nephrogenic ascites (Table 3). The ascitic fluid analysis was suggestive of high SAAG and low protein. In this group with an unknown etiology (n=11), four died, two were lost to follow-up, and the remaining five patients were re-evaluated at the end of three months and no specific etiology of ascites could be identified (Figure 1).

**Table 3:** Characteristics of patients with nephrogenic ascites (N=11). CKD: chronic kidney disease; ESRD: end-stage renal disease; MHD: maintenance hemodialysis; HD: hemodialysis; AV fistula: arteriovenous fistula; SAAG: serum ascites-albumin gradient

Variables	Values
Males, n	8
Age (years), mean±SD	45.3±12.8
Diabetes mellitus, n	4
Hypertension, n	9
Coronary artery disease, n	1
Alcohol, n	3
Hypertensive crisis, n	3
Fever, n	2
Weight loss, n	1
Jaundice, n	1
CKD duration (months), mean±SD	25.1±29.3
ESRD on MHD, n	6
Duration of dialysis (months), mean±SD	10.1±15.9
AV fistula, n	3
HD twice weekly, n	5
HD thrice weekly, n	1
Ascitic fluid lymphocytes (%), mean±SD	60.5±37.4
Ascitic fluid protein (g/dL), mean±SD	2.2±1.5
Ascitic fluid SAAG (g/dL), mean±SD	1.4±0.4

**Figure 3:**
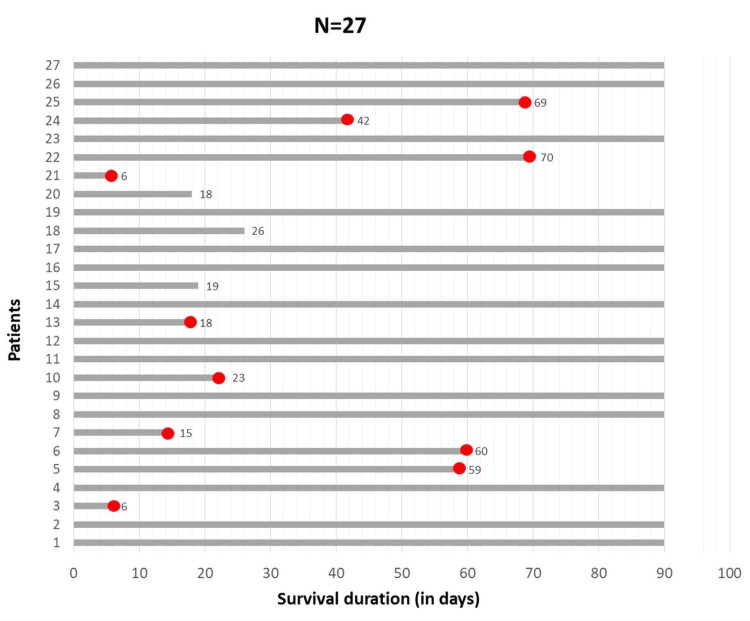
Follow-up data of 27 patients at the end of 90 days.

Of the 40 patients, 13 (32.5 %) were lost to follow-up after three months. Among the remaining 27 patients, 14 (51.8 %) survived until the end of the first three months (Figure 3); five patients showed significant improvement in ascites and related symptoms. Among these five patients, one had switched over to continuous ambulatory peritoneal dialysis (CAPD), one had an increased frequency of hemodialysis sessions, one had an increased frequency of large-volume paracentesis, and the remaining two patients improved only with salt and fluid restriction. In contrast, six patients who had an increased dosage of diuretic administration did not show any improvement at the end of three months.

## Discussion

Progressive CKD (Grade G3a and above) is associated with several complications that are associated with severe adverse clinical outcomes, such as cardiovascular events and renal failure requiring renal replacement therapy (RRT) [21]. A scantly discussed complication remains the development of ascites in CKD patients as the disease advances. The current study aimed to determine the prevalence and etiology of ascites, focussing on the rare entity, nephrogenic ascites, and the 90-day mortality in patients with ascites. The prevalence of ascites among patients with CKD in our study was estimated at 11.3%. Despite extensive workup, the etiology of ascites remained unknown in 11/40 (27.5%) patients, and they were considered to have nephrogenic ascites. In comparison, a study from North India reported nephrogenic ascites in 23/150 (15%) [16].

Normally, the peritoneal cavity contains approximately 50-75 mL of fluid, an ultrafiltrate of plasma [22]. Fluid formation increases due to increased venous hydrostatic pressure, decreased plasma oncotic pressure, increased permeability of peritoneal capillaries, or lymphatic obstruction. Liver cirrhosis is the most common cause, accounting for approximately 80% of all cases worldwide [23]. According to the International Ascites Club guidelines, ascites is clinically graded as grade 1 (detectable only by ultrasonography), grade 2 (moderate ascites causing symmetrical distension), and grade 3 (large ascites causing marked abdominal distension) [24]. Elevated SAAG (≥ 1.1 g/dL) is usually associated with increased portal pressure, as in cases of liver cirrhosis or congestive cardiac failure. A low SAAG suggests other causes of normal portal pressure, such as infection or malignancy [25]. While SAAG is the first index to classify ascites, total ascitic fluid protein (AFP) is a second-line test, first to differentiate spontaneous (SBP) from secondary bacterial peritonitis, second to predict those at a high risk of SBP based on a low AFP and finally, to suggest heart failure in patients with high SAAG ascites [19,26].

The association between ascites and CKD was first reported in 1970, 10 years after the introduction of MHD in the United States [4,5]. Since then, the entity of ascites in CKD has been addressed in a few case reports, case series, and reviews. Gotloib and Servadio showed that ascitic volume in these patients was commonly out of proportion to that of pedal edema and was reversed after successful kidney transplantation or by fluid restriction [10]. In 1987, Gluck and Nolph reviewed 138 patients with ascites and ESRD [7]. Ascitic fluid was positive for acid-fast bacilli in one case. All the fluid samples tested negative for malignant cytology. This study highlighted the importance of an extensive investigation of ascites in patients with ESRD.

In 1994, Cintin and Joeffe described ascites in two patients with ESRD undergoing hemodialysis [14]. Both patients were initially treated with fluid restriction, ultrafiltration, and vigorous HD. However, they did not improve despite these measures, and subsequently underwent renal transplantation, after which one of the patient’s ascites resolved. None of our patients underwent transplantation, and only the frequency of HD increased with fluid restriction. Hammond and Takkiyuddin reported another case of ascites in an ESRD patient on MHD for 18 months who succumbed six months after the initial diagnosis of nephrogenic ascites [6]. Ascitic fluid showed low SAAG and high protein levels. 

Males comprised nearly three-fourths of the study population. This is very similar to Sethi et al. (M: F, 3:1), Gluck and Nolph (M: F, 2:1), and Mauk et al. (all nine males) [7,12,16]. Conversely, Han et al. reported equal proportions of both sexes [9]. Among the patients with ascites, the most common etiology of underlying CKD was hypertension, followed by diabetes mellitus (Table 2). Another study from the Indian subcontinent also reported similar findings [16]. Older studies have revealed that chronic glomerulonephritis is the most common cause of CKD [7].

In the current study, patients with ascites were on MHD for a mean of 9.3±15.5 months, significantly longer than those without ascites. This was relatively lesser in comparison to Han et al.(12/16 patients with a mean of 20 months), Gluck and Nolph (18 months before and 69 months after initiation), and Mauk et al.(7/9 patients with ascites 36 months after initiation of MHD) [7,9,12]. Thus, it is difficult to establish a temporal association between ascites onset and dialysis duration. The mean duration of ascites in our study population was 35.6 days (1.18 months), which was significantly shorter than that reported by Gluck and Nolph (11 months) and Han et al. (four months) [7,9].

Four patients had hypothyroidism, which has been described in several studies as the etiology of ascites [27,28]. Many patients with ascites had more than one identifiable etiology; hence, they were allocated to mixed ascites or the multiple etiologies subgroup (Figure 1). In comparison, 118/138 (85.5%) patients in the study by Gluck and Nolph did not have an underlying etiology; 47 patients underwent peritoneal biopsy, with pathological findings noted in 21 patients [7]. Ten (62.5%) patients in the study by Han et al. [9] and all patients in the study by Mauk et al. underwent peritoneoscopy [12]. None of our patients underwent this procedure, which remains a limiting factor. Interventional procedures during the study period were limited due to the ongoing COVID-19 pandemic.

Traditionally, nephrogenic ascites has been described as either exudative or low SAAG. Cardiac, hepatic, malignant, and infectious diseases should be ruled out. Among the 11 patients with an unknown etiology, 10 were in the high-SAAG group, and none belonged to the traditional exudative/low-SAAG group. Eight of our patients with high SAAG ascites had mixed ascites. Thirteen patients in the low-SAAG group had more than one cause of ascites, which could not be referred to as mixed ascites (Figure 1). Nayak-Rao reported three cases of nephrogenic ascites: one with high SAAG and two with low SAAG [8]. Gunal described six patients with nephrogenic ascites, all of whom had a major component of right-sided heart failure, thus, by definition, a high-SAAG ascites, but this test had not been calculated [29]. Multiple case reports in the last five years have shown that nephrogenic ascites are predominantly low-SAAG and high-protein compared to our patients with predominant high-SAAG ascites [30,31].

Hence, these findings are quite different from those of older studies (before SAAG came into vogue), which mainly described the exudative nature of nephrogenic ascites. Glück and Nolph reported 118/138 patients had high AFP (>2.5 g/dL) [7]. Similarly, all nine cases in the series by Mauk et al. had exudative high-protein ascites [12]. SAAG values were available for only 5/16 (31.2%) patients in the study by Han et al., with a mean SAAG of 0.99 and mean AFP of 4.0 g/dL (low SAAG and high protein) [9]. In Sethi et al.’s study, all 23/150 (15.3%) patients had low SAAG ascites, in contrast to the current study, where only one patient had a similar finding [16]. Thus, compared to several previous studies, our study highlights the heterogenicity of ascitic fluid characteristics in CKD patients, which can be explained by the multiple mechanisms involved in the development of ascites in these patients. Interestingly, patients in whom hypothyroidism was thought to be a contributing factor also had variable SAAG and ascitic fluid protein values. This is supported by the findings of several studies wherein myxedema ascites of low SAAG and high protein type may have varying SAAG and AFP values [28,32].

Of the 40 patients with ascites, 13 were lost to follow-up after discharge. Among the 27 patients followed up, 13 (nearly 50%) died within three months of enrolment in the study. This reiterates the fact that ascites development in patients with CKD has a poor prognosis and high mortality, as described in previous studies. In the study by Gluck and Nolph, one-third of 118 patients died 10.7 ± 7.0 months after the onset of ascites [9]. Four (44.4%) patients described by Mauk et al. died with an average survival of 7.3 months from the onset of ascites [9]. Sethi et al. reported a mortality rate of 17.4% [9]. In the current study, infections and comorbid illnesses contributed to the mortality. Massive ascites was seen in nine. Catheter-related bloodstream infection (n=5), spontaneous bacterial peritonitis (n=1), necrotizing fasciitis (n=1), septic shock (n=3), COVID-19 pneumonia (n=1), disseminated tuberculosis (n=1), bilateral pyelonephritis (n=1), and antituberculous therapy-related hepatitis (n=1) contributed towards the mortality. Delays in arrival to the hospital due to transport restrictions constituted the biggest detriment in the treatment of infections. Heart failure with reduced ejection fraction and coexisting cirrhosis contributed to the poor improvement in patient symptoms even with fluid restriction, diuretics, and daily hemodialysis.

Strengths and limitations

This prospective study had a larger sample size than other Asian studies. We estimated the prevalence of significant ascites in CKD patients. A follow-up period of three months was also performed to assess mortality. As this was a single-center study of Nephrology and Transplantation services, the patient population may not apply to other parts of India. Liver elastography was not performed. Invasive procedures such as peritoneoscopy, liver biopsy, and right heart catheterization were not performed. There was a significant loss to follow-up.

## Conclusions

In the current study, the prevalence of ascites in CKD patients was 11.3%. Eleven patients had nephrogenic ascites but did not fit the usual pattern of low SAAG/high protein, which has usually been described in the literature, and the presence of ascites was associated with a significantly longer maintenance hemodialysis duration. Most patients with CKD and ascites have an identifiable etiology, highlighting the importance of early diagnosis and treatment. The significant contributing factors include cardiac failure and hypothyroidism. The study also highlights the importance of ascitic fluid SAAG and AFP analysis, which may help unmask multiple etiologies contributing to ascites with heterogeneous characteristics. Ascites in CKD had a dismal prognosis with a high mortality rate, as reflected by the follow-up of the patients in our study.
